# Novel antitenascin antibody with increased tumour localisation for Pretargeted Antibody-Guided RadioImmunoTherapy (PAGRIT^R^)

**DOI:** 10.1038/sj.bjc.6600818

**Published:** 2003-04-01

**Authors:** R De Santis, A M Anastasi, V D'Alessio, A Pelliccia, C Albertoni, A Rosi, B Leoni, R Lindstedt, F Petronzelli, M Dani, A Verdoliva, A Ippolito, N Campanile, V Manfredi, A Esposito, G Cassani, M Chinol, G Paganelli, P Carminati

**Affiliations:** 1 Immunology Department, Sigma Tau SpA R&D, Via Pontina Km 30.400, 00040 Pomezia, Rome, Italy; 2 Tecnogen SCpA Località La Fagianeria, Piana di Monte Verna, 81015 Caserta, Italy; 3 Division of Nuclear Medicine, European Institute of Oncology, Via Ripamonti, 435, 20141 Milan, Italy

**Keywords:** antibody pretargeting, monoclonal antibody, tenascin

## Abstract

The Pretargeted Antibody-Guided RadioImmunoTherapy (PAGRIT) method is based on intravenous, sequential administration of a biotinylated antibody, avidin/streptavidin and ^90^Y-labelled biotin. The hybridoma clone producing the monoclonal antitenascin antibody BC4, previously used for clinical applications, was found not suitable for further development because of the production of an additional, nonfunctional light chain. In order to solve this problem, the new cST2146 hybridoma clone was generated. The monoclonal antibody ST2146, produced by this hybridoma, having the same specificity as BC4 but lacking the nonfunctional light chain, was characterised. ST2146 was found able to bind human tenascin at an epitope strictly related, if not identical, to the antigenic epitope of BC4. It showed, compared to BC4, higher affinity and immunoreactivity and similar selectivity by immunohistochemistry. Biodistribution studies of biotinylated ST2146 and three other monoclonal antitenascin antibodies showed for ST2146 the highest and more specific tumour localisation in HT29-grafted nude mice. On the overall, ST2146 appears to be a good alternative to BC4 for further clinical development of PAGRIT.

The specificity of tumour therapies is often a limiting factor in determining the success of a treatment. In fact, the onset of toxic effects and the reduced tolerability of certain anticancer agents limit their use and affect the quality of life of patients.

The reduction of toxicity is directly related to the selectivity of treatment for cancer cells. Monoclonal antibodies (Mabs) can be used for specific targeting of tumours, and when combined with the avidin/biotin amplification system they constitute a powerful and selective way to deliver active moieties to the tumour site.

A three-step Pretargeted Antibody-Guided RadioImmunoTherapy (PAGRIT) approach for systemic and locoregional treatment of brain tumours was previously shown to be safe and to produce clinical benefits to patients (Cremonesi *et al*, 1999; Paganelli *et al*, 1999,^2001^; Grana *et al*, 2002).

PAGRIT is based on intravenous, sequential administration of biotinylated monoclonal antibodies, avidin/streptavidin and ^90^yttrium (^90^Y)-labelled biotin. The selectivity of the three-step pretargeting approach relies on the use of a biotinylated monoclonal antitenascin antibody. Tenascin-C is an extracellular matrix protein, which is abundant in the stroma of several solid tumours (Natali *et al*, 1991; Mackie, 1997). It is a hexameric glycoprotein that can undergo alternative splicing resulting in large (up to 320 kDa monomer) or small (220 kDa monomer) isoforms. The large tenascin variant, which is preferentially expressed in malignant tissues, has been shown to exert anti-adhesive effects thus favouring tumour metastasis (Jahkola *et al*, 1998; Ghert *et al*, 2001). Moreover, tenascin expression, similar to vascular endo thelial growth factor/vascular permeability factor expression, is spatially and temporally related to neovascularisation (Zagzag and Capo, 2002). An immunosuppressive activity has also been reported for the large tenascin domain FnIIIA1A2 on activation-induced T-lymphocyte proliferation and cytokine production *in vitro* (Puente Navazo *et al*, 2001). These features of tumour-associated tenascin make this protein a good candidate for tumour targeting. Moreover, the targeting of extracellular matrix, compared to the targeting of tumour cell surface antigens, exhibits the advantage of being unaffected by antigen modulation of tumour cells, thus representing an ideal way for antitumour therapy. The role of the biotinylated antitenascin antibody is to display biotins at the tumour site in order to mediate subsequent streptavidin and ^90^Y-biotin accumulations.

The monoclonal antitenascin antibodies BC4 and BC2 described by Siri *et al* (1991) and Balza *et al* (1993) have been previously used, with success, in both systemic and topical, pretargeted or direct therapeutic settings in patients with brain tumours (Riva *et al*, 1997; Paganelli *et al*, 1999,^2001^; Grana *et al*, 2002). However, both BC2 and BC4 hybridoma clones were found unsuitable for process development because of the production of an additional, nonfunctional light chain (most likely of parental myeloma origin) whose increased level of expression in the scaled-up cultivation prevented a large-scale antibody purification (Vola *et al* (1993) and results from our laboratories). In order to generate a new hybridoma cell clone with the specificity of BC4 but lacking the expression of nonfunctional light chains, mice were immunised with the recombinant EGF-like repeat fragment of tenascin previously shown to contain the BC4 antigenic epitope (Balza *et al*, 1993). Hybridoma supernatants were screened on purified tenascin and the antibody ST2146 was selected. Both *in vitro* and *in vivo* characterisations of ST2146 showed that it might be a good alternative to BC4.

## MATERIALS AND METHODS

### Hybridomas and monoclonal antibodies

BC4 and BC2 hybridomas were kindly provided by Dr Luciano Zardi (Laboratory of Cell Biology, Istituto Nazionale per la Ricerca sul Cancro, Genova, Italy) and the related monoclonal antibodies, both of IgG1/k isotype, were purified from the hybridoma culture supernatants by protein A chromatography followed by hydroxylapatite chromatography as described by Vola *et al* (1993). ST1897 (IgG1/k isotype) was obtained from Wistar Institute, Philadelphia, PA, USA (original code 300-2; Herlyn *et al*, 1991). In order to generate new hybridoma cell clones with the specificity of BC4, Balb/c mice were immunised with pTn28 *E. coli* phage lysate. pTn28 is a λgt11 recombinant clone encoding a fragment of the EGF-like repeats of human tenascin previously shown to contain the BC4 epitope (Balza *et al*, 1993). pTn28 immunised splenocytes were fused to Sp2/0Ag14 nonproducing myeloma cells (obtained from ECACC, Italy) by standard methods (Cianfriglia *et al*, 1986). The hybridoma population was screened by ELISA on purified SK-MEL-28 (human melanoma cell line obtained from Interlab Cell Line Collection (ICLC), Genova, Italy) tenascin. Tenascin-specific hybridomas were cloned by limiting dilution two times in FCS containing medium and three times in protein-free medium (Animal Derived Component Free Medium HyClone, HyQ^R^ Perbio). The cST2146 subclone was selected for the production of the cST2146 Master Cell Bank (MCB) and Working Cell Bank (WCB). MCB samples were deposited at the ICLC, Genova, Italy for patent purposes and the patent application 60/359.299 was submitted in US.

### ST2146 production and purification

The production of Mab ST2146 was performed by cultivation of cST2146 hybridoma cells in protein-free medium, in a 2 l perfusion bioreactor (MD2, B Braun). The stability of cST2146 Post Production Cell Bank (PPCB) was confirmed by FACS analysis and by limiting dilution. The ST2146 isotype was determined by the use of a commercial kit (Mouse-hybridoma subtyping kit, Boehringer Mannheim, Monza, Italy). Purification was performed by three chromatography steps. The 10 × concentrated bioreactor harvest was loaded on MEP HyperCell column (BioSepra, Cergy, Saint Christopher, France) in 50 mM TRIS pH 8.0. The column was washed with five bed volumes of binding buffer and the retained material eluted by 50 mM sodium acetate, pH 4.0. The eluted material, adjusted to pH 8.0 with 1 M TRIS, was loaded on a Q-Sepharose-XL column (Pharmacia, Milan, Italy) equilibrated with 50 mM TRIS, pH 8.0; elution was performed with 50 mM sodium acetate, 250 mM NaCl, pH 6.0. The final SP-Sepharose-XL chromatography was performed by loading the material eluted from Q-Sepharose and adjusting pH to 5.0, and then eluting with 0.1 M sodium bicarbonate, 0.5 M NaCl, pH 8.5, a condition useful for the next step of antibody biotinylation.

### ST2146 deglycosylation

To investigate the heavy-chain heterogeneity of ST2146, the Mab, at a concentration of 1 mg ml^−1^, was digested with 1.5 U ml^−1^ of sialidase (Sigma, Milan, Italy) in 10 mM NaHPO_4_, 150 mM NaCl, pH 6.4, at 37^o^C for 24 h. The digested and not digested samples were subjected to electrophoresis on 12% polyacrylamide slab gels and stained with Coomassie Brilliant Blue.

### Hydroxylapatite chromatography

The separation of the three immunoglobulin variants of BC4 was done essentially as described by Vola *et al* (1993). Briefly, hydroxyl apatite chromatography analysis was carried out by loading 100–300 *μ*g of protein A-purified BC4 or ST2146 onto a 1 ml Macro-Prep Ceramic Hydroxylapatite Type II (20 *μ*m) column, 10 × 6 mm^2^ (BIO-RAD, Milan, Italy) equilibrated at a flow rate of 0.5 ml min^−1^ with 10 mM sodium phosphate, pH 6.8. Elution was performed with a concentration gradient of phosphate buffer (10–150 mM in 50 min).

### Western blot analysis

Human tenascin purified from SK-MEL-28 cell supernatant as previously described (Saginati *et al*, 1992), purified A–D fragment and pTn28 phage lysate (kindly provided by Dr L Zardi) were run on 12% reducing, SDS–PAGE and transferred onto a nitrocellulose membrane by electroblotting. After protein transfer, the membrane was incubated overnight at +4°C in 0.1 M TRIS, 0.15 M NaCl, 5% dried milk, to block nonspecific binding. After five washings with the same blocking buffer, the membrane was incubated for 1.5 h at room temperature with BC4, BC2 or ST2146 diluted in PBS at 5 *μ*g ml^−1^. After washings, a goat anti-mouse IgG-alkaline phosphatase conjugate (Amersham, Milan, Italy), diluted 1 : 1000 in blocking buffer, was used incubating for 1 h at room temperature; after three washings with 0.1 M TRIS, 0.1 M NaCl, 0.08 M MgCl_2_, the chromogenic substrate solution, consisting of 0.330 mg ml^−1^ nitro blue tetrazolium (Sigma, Milan, Italy), 0.165 mg ml^−1^ 5-bromo-4-chloro-3-indolyl phosphate in blocking buffer, was employed.

### Immunoreactivity and competitive ELISA

Immuno™ MAXISORP 96-well plates (Nunc) were coated at 4°C overnight with 100 *μ*l well^−1^ of tenascin solution at 5 or 0.5 *μ*g ml^−1^ in PBS, pH 7.2. Tenascin was purified from the culture supernatant of the melanoma cell line SK-MEL-28 as described previously. After coating, the plates were washed once with PBS, 0.1% Tween 20 (washing buffer) and blocked with 300 *μ*l well^−1^ of PBS, 0.1% Tween 20, 1% BSA (blocking and diluting buffer) for 2 h at room temperature. Plates were used immediately or dried and frozen at −20°C. For immunoreactivity analysis the plates were incubated with serial dilutions of antitenascin Mabs (100 *μ*l well^−1^) for 1.5 h at 37°C. Each dilution was assayed in duplicate. After three washings, the plates were incubated for 1.5 h at 37°C with 100 *μ*l well^−1^ of anti-mouse IgG (Fc-specific) alkaline phosphatase-conjugated (SIGMA, St. Louis, IL, USA) diluted 1 : 1000 in blocking buffer. The plates were then washed four times, incubated with 200 *μ*l well^−1^ of pNPP (SIGMA, St. Louis, IL, USA) for 30 min at 37°C, stopped with 100 *μ*l well^−1^ of 3 M NaOH and read with an ELISA spectrophotometer (SEAC Sirio S) at 405 nm. For competitive ELISA, the biotinylated monoclonal antibody ST2146 (IgG2b) was mixed with increasing concentrations of ST2146, BC4 (IgG1), or a not related IgG1 (SIGMA, St. Louis, IL, USA) as competitors; 100 *μ*l well^−1^ of this mixture was plated on tenascin-coated plates. Optimal concentrations of tenascin and biotinylated ST2146 were determined in preliminary experiments. After 2 h at room temperature, the plates were washed three times, incubated for 30 min at room temperature with 100 *μ*l well^−1^ of horseradish peroxidase–streptavidin (Amersham, Milan, Italy) diluted 1 : 1500 in blocking buffer, washed again and incubated for 30 min at room temperature with 200 *μ*l well^−1^ of TMB substrate (SIGMA, St. Louis, IL, USA). The reaction was stopped with 100 *μ*l well^−1^ of 0.5 M H_2_SO_4_ and the plate read with an ELISA spectrophotometer (SEAC Sirio S) at 405 nm.

### BiaCore analysis

BIAcoreX instrument, CM5 sensorchip, HBS buffer (10 mM Hepes, 0.15 mM NaCl, 3.4 mM EDTA and 0.005% surfactant P20, pH 7.4), amine coupling kit (NHS, *N*-hydroxysuccinimide; EDC, *N*-ethyl-*N*′dimethylaminopropylcarbodiimide) and ethanolamine were all obtained from Biosense (Milan, Italy). Tenascin was immobilised on a CM5 sensor chip as follows. A continuous flow of HBS at 5 *μ*l min^−1^ was maintained and the carboxymethylated dextran-coated surface was activated by a 7-min injection of a solution containing 200 mM EDC and 50 mM NHS followed by the injection of tenascin (82 *μ*g ml^−1^ in 10 mM sodium acetate brought to pH 2.3 with HCl). Capping of unreacted sites was achieved by injecting 1 M ethanolamine, pH 8.5, for 7 min. Excess tenascin was washed off with repeated injections of 100 mM NaOH. The final immobilisation response was 3200 resonance units (RU). Sensorgrams were generated by the injection of Mabs (at five concentrations ranging from 3.9 nM to 500 nM) in HBS at a flow rate of 30 *μ*l min^−1^. The binding was not affected by the flow rate (tested between 5 and 30 *μ*l min^−1^) suggesting no mass transport limitation for the Mab binding. Kinetic data were collected in duplicate for each concentration of Mab. The association and dissociation times were 60 and 120 s, respectively. The chip was regenerated by injection of repeated pulses of 100 mM NaOH until the difference between the baseline before and after injection was less than 10 RU. Biosensor data were prepared, modelled and fitted by means of the BIA evaluation 3.1 software. The evaluation of the data was done using a bivalent-analyte model with simultaneous determination of association and dissociation constants. The quality of the fitted data was evaluated by comparison between calculated and experimental curves (residual values) and by the magnitude of the *χ*^2^ parameter.

### Immunohistochemistry

Immunohistochemistry studies were conducted on tissue array slides (SuperBioChips Laboratories, Korea) including various human tumours and normal organs. Slides were processed according to the manufacturer's instructions and binding was revealed by the use of the Vectastain ABC kit (Vector Laboratories, Burlingame, CA, USA). Briefly, after deparaffination and hydration, the tissue sections were blocked with normal blocking serum. Slides were then incubated overnight at 4°C with BC4 or ST2146 at 10 and 2 *μ*g ml^−1^ in PBS, respectively. After three washings with PBS and incubation for 30 min with a biotinylated goat anti-mouse antibody, the slides were incubated for 30 min with the avidin–biotin–peroxidase complex. After three washings with PBS, DAB substrate was added and the reaction was stopped after 2 min by washing in tap water. Counterstaining was performed by Mayer's haematoxylin for 10 s. Negative controls included slides incubated with an isotype-matched, nonrelevant antibody or with only the second antibody.

### Radiolabelling of Mabs and animal study

Antibodies at 200–400 *μ*g ml^−1^ were mixed with chloramine T (Sigma, Milan, Italy) at a final concentration of 2–10 *μ*g ml^−1^ and with 1–4 *μ*Ci (37–148 kBq) of sodium ^125^iodine (New Life Science, Milan, Italy) per microgram of antibody. After 5–10 min at room temperature, the reaction mixture was buffer exchanged three times with 1.0 ml of sterile PBS on Vivaspin membrane, cut off 30 000 (Sartorius, Germany) to remove free iodine. Immunoreactivity of radiolabelled antibodies was checked by ELISA as described in the ‘Immunoreactivity and competitive ELISA’ section under Materials and Methods on plates coated with tenascin at 1.0 *μ*g ml^−1^. Balb/c nu/nu mice (Charles River, Calco, Como, Italy) were transplanted subcutaneously (s.c.) with 5 × 10^6^ HT29 human colon carcinoma cells (obtained from DSMZ, Germany) in 0.1 ml of PBS. After 15 days, mice were randomised into treatment groups (five animals/group) and intravenously (i.v.) injected with 2 *μ*g of ^125^I-radiollabeled antibodies (1–2 × 10^6^ c.p.m. animal^−1^). Normal mouse immunoglobulins (Sigma, Milan, Italy) were used as a control. After 48 and 72 h from injection mice were killed by cervical dislocation and blood, spleen, kidney, liver and tumour mass collected. Each tissue was weighted and counted in a *γ*-counter (Packard, Canberra). Data were expressed as the percentage of injected dose g^−1^ of tissue (% i.d. g^−1^). The data were statistically analysed by the Student's t method.

The care and husbandry of animals were in accordance with the European Directive 86/609 and with Italian legislation.

## RESULTS

### Generation and *in vitro* characterisation of ST2146

**Figure 1:**
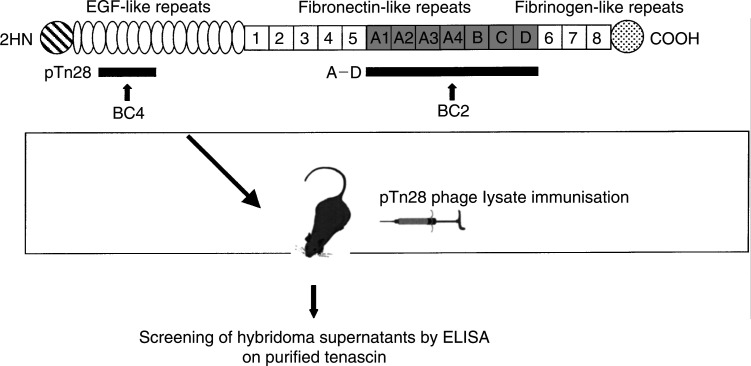
Schematic representation of human tenascin-C, pTn28 and A–D recombinant fragments and strategy to generate a BC4-like antibody.

In order to generate a BC4-like monoclonal antibody, hybridomas were produced from mice immunised with the recombinant pTn28 phage lysate encoding the EGF-like repeats of human tenascin previously shown to contain the BC4 antigenic epitope. Figure 1

shows the schematic representation of human tenascin, the related recombinant pTn28 and A–D antigenic fragments, as well as the strategy used to generate a BC4-like antibody. ST2146 was purified from the bioreactor protein-free conditioned medium by three chromatography steps and was found to be of the IgG2b/k isotype.

#### Biochemical characterisation

**Figure 2:**
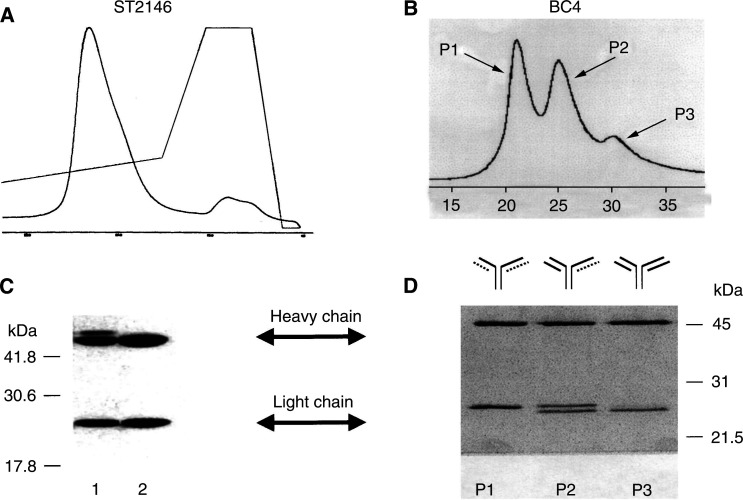
Hydroxylapatite chromatography of ST2146 (**A**) and BC4 (**B**). SDS–PAGE analysis under reducing conditions of ST2146 not digested (**C**, lane 1) or digested with sialidase (**C**, lane 2) and BC4 peaks 1, 2 or 3 from hydroxylapatite chromatography (**D**, P1, P2 and P3, respectively). *Note*: The dotted chain was found to be not functional. Therefore, divalent BC4 corresponds only to peak 3.

The homogeneity of ST2146 was evaluated by hydroxylapatite chromatography which showed a single peak for ST2146 as opposed to three peaks observed for BC4 (Figure 2

, panels A and B). By reducing SDS–PAGE analysis, the hydroxylapatite peak of ST2146 proved to be homogeneous for light-chain composition while a certain degree of heterogeneity for the heavy chain was apparent (Figure 2, panel C, lane 1). This observation was consistent with the variability in *O*-linked glycosylation previously reported for the murine IgG2b/k isotype (Kim *et al*, 1994). Consistency in the pattern of the ST2146 heavy-chain bands was observed in three different batches obtained from FCS-containing or protein-free culture media (data not shown). The glycosidic nature of the ST2146 heavy-chain variability was confirmed by digesting the antibody with sialidase, which caused the disappearance of the higher molecular weight band (Figure 2, panel C, lane 2). The SDS–PAGE analysis of the hydroxylapatite peaks of BC4 showed the heterogeneous composition of this Mab, with peak 1 containing a light chain of higher molecular weight compared to the light chain of peak 3 (Figure 2D, lanes P1 and P3), and peak 2 composed of both high and low molecular weight light chains (Figure 2D, lane P2).

#### Specificity and immunoreactivity

**Figure 3:**
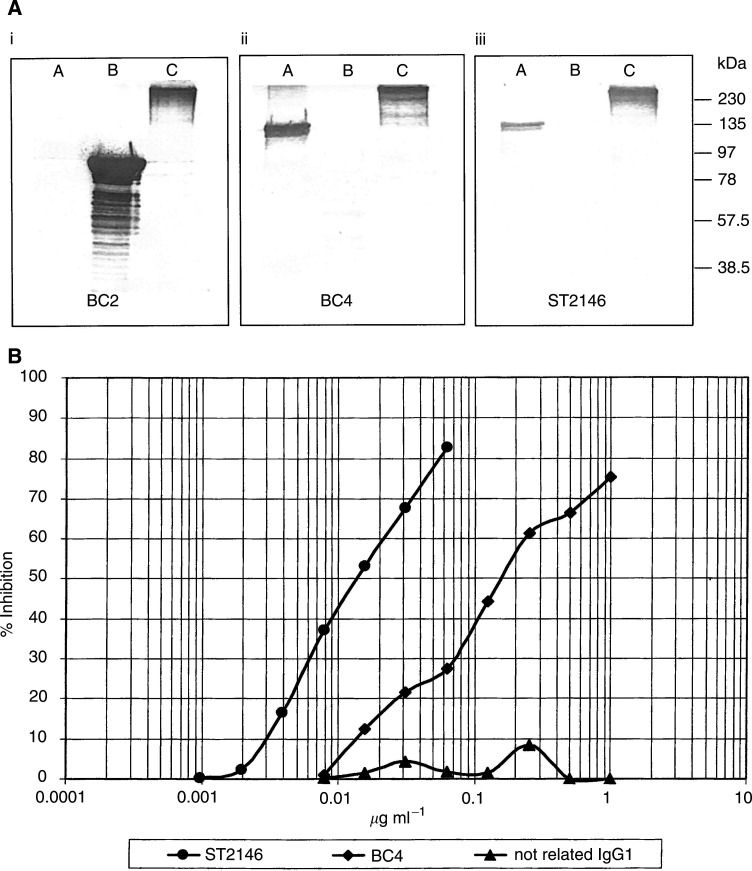
(**A**) Western blot analysis of BC2 (i), BC4 (ii) and ST2146 (iii) antibodies. A: pTn28; B: A–D fragment; C: tenascin. (**B**) Competitive ELISA. Biotinylated ST2146 was mixed with increasing concentrations of ST2146, BC4 or a not related IgG1 as competitors and plated on tenascin-coated plates. Binding was measured after addition of horseradish peroxidase–streptavidin and related chromogenic substrate.

**Figure 4:**
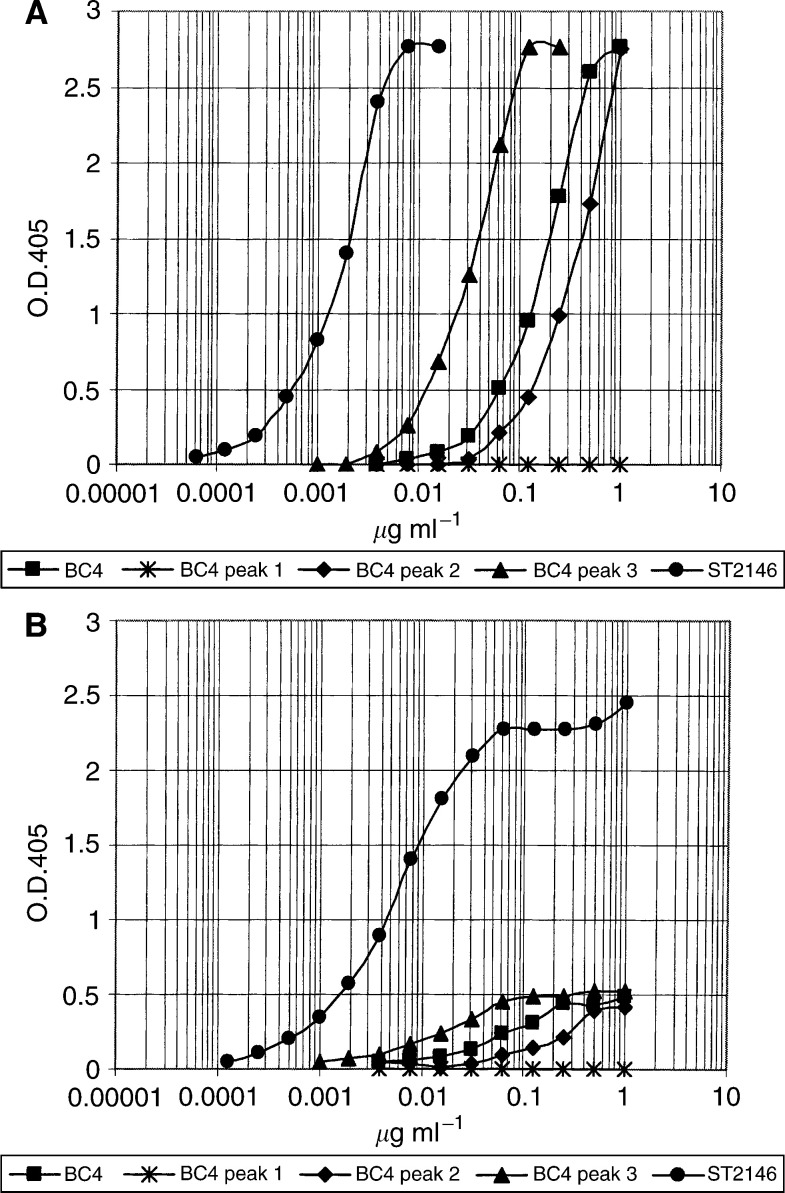
ELISA plates coated with tenascin at 5 *μ*g ml^−1^ (**A**) or 0.5 *μ*g ml^−1^ (**B**). BC4 peaks 1–3 were obtained by subjecting the protein A eluted BC4 to hydroxylapatite chromatography as shown in Figure 2B.

ST2146 binds to human tenascin at an epitope strictly related, if not identical, to the antigenic epitope of BC4, as shown by Western blotting in Figure 3A

, which indicates that BC4, BC2 and ST2146 are all recognising tenascin (lane C of panels i–iii) with BC4 and ST2146 reactive with the EGF-like repeats region of tenascin (pTn28 in lane A of panels i–iii) and BC2 reactive with the A–D region (lane B of panels i–iii). Competition binding data in Figure 3B indicate a lower immunoreactivity of BC4 compared to ST2146 as the amount of BC4 inhibiting 50% binding of ST2146 to tenascin is about 10 times that of ST2146. This result was also confirmed by direct ELISA. Figure 4

shows that the amount of ST2146 giving 1.0 o.d. by ELISA in condition of optimal tenascin coating (panel A), is approximately 20 times less than the amount of divalent BC4 (peak 3 of Figure 2B) and approximately 100 times less than that of BC4 (pool of peaks 1–3). This difference in immunoreactivity is dramatically amplified in conditions of antigen limitation, as shown in panel B of Figure 4, where only ST2146 maintains significant immunoreactivity.

#### Affinity

**Figure 5:**
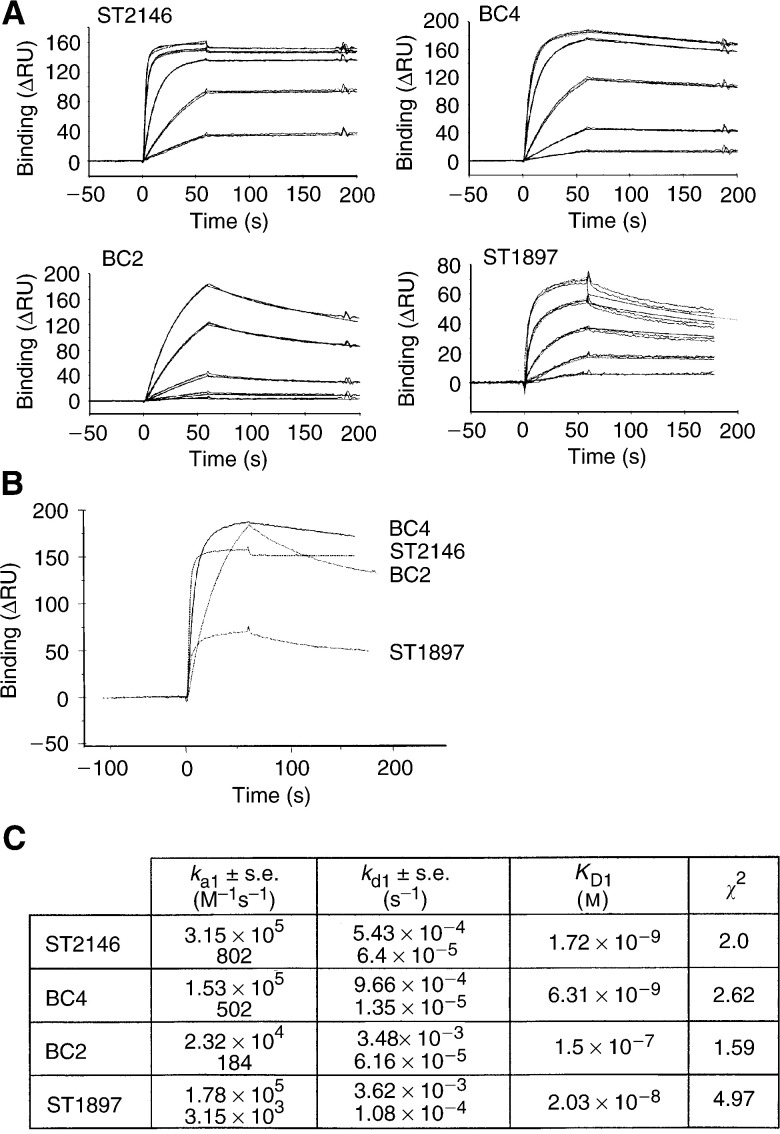
BiaCore analysis of ST2146, BC4, BC2 and ST1897 on tenascin-coated chip. (**A**) Sensograms of indicated Mabs. All antibodies were injected at concentrations of 500, 250, 62.5, 15.6 and 3.9 nM for 60 s. The dissociation rates of the Mabs were determined over a time of 120 s. (**B**) Comparison of the binding and dissociation of the indicated Mabs at an injected concentration of 500 nM. (**C**) *k*_a1_, *k*_d1_, *K*_D1_ and *χ*^2^ of the fitted curves.

The affinities of ST2146, BC4, BC2 and ST1897 were evaluated by BIAcore on a tenascin-coated chip. The sensorgrams as well as the kinetic values of Figure 5

indicate for ST2146 the most rapid antigen association followed by ST1897, BC4 and BC2. The dissociation rate of ST2146 was the lowest followed by BC4, ST1897 and BC2. The antigen binding kinetics indicate for ST2146 the highest affinity for the human tenascin within the panel of antibodies tested. The higher number of RU observed with BC4 and BC2 might be consistent with the presence of a significant fraction of monovalent immunoglobulins in BC4 and BC2 antibody batches which might lead to an overall higher amount of bound antibody compared to divalent immunoglobulins ST2146 and ST1897.

#### Immunohistochemistry

**Figure 6:**
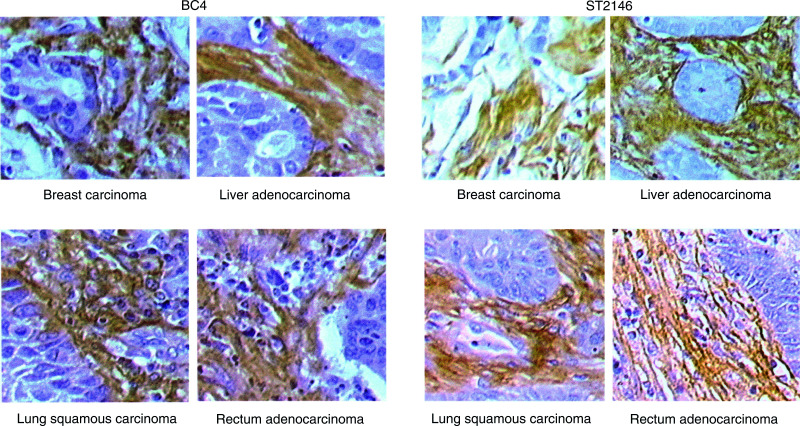
Immunohistochemistry on serial sections of breast, liver, lung and rectum carcinomas with BC4 and ST2146 antibodies. × 20 magnification.

Immunohistochemistry with ST2146 was performed in parallel to BC4 on tissue array slides that included 20 different tumours and more than 60 normal tissues. BC4 and ST2146 showed similar selectivity. In fact, both antibodies strongly react with the stroma of several solid tumours including breast, liver, lung and rectum carcinomas reported as examples in Figure 6

. ST2146 and BC4 also detect tenascin in the interstitium of several normal tissues (data not shown) as previoulsy published for BC4 (Natali *et al*, 1991).

### Biodistribution

**Figure 7:**
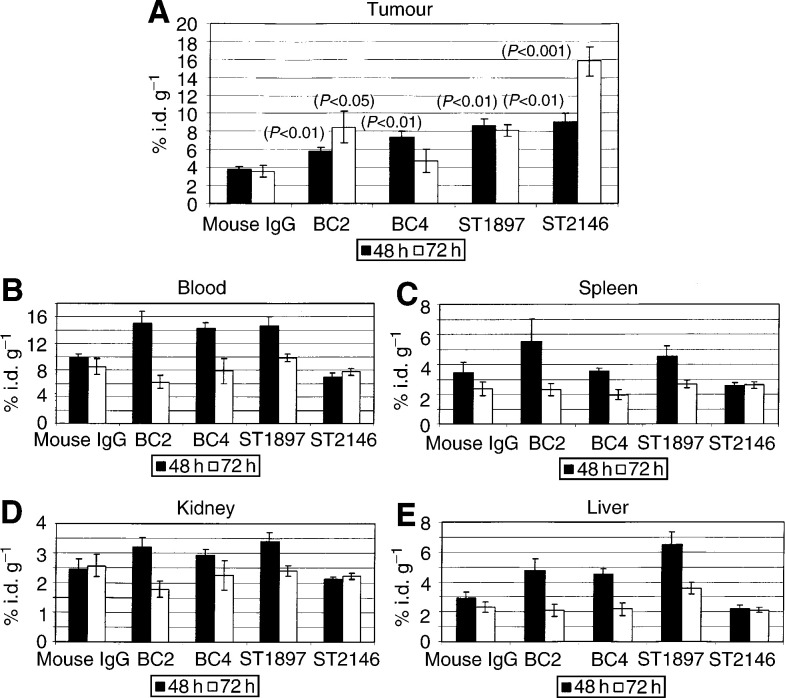
Biodistribution of ^125^I-labelled ST2146, BC4, BC2, ST1897 and normal mouse IgG. Data represent the mean values, ±standard error, of the % of injected dose per gram of tissue (%i.d. g^−1^) from five animals at 48 or 72 h. *P*-values were obtained *vs* normal mouse IgG by Student's *t*-test.

**Figure 8:**
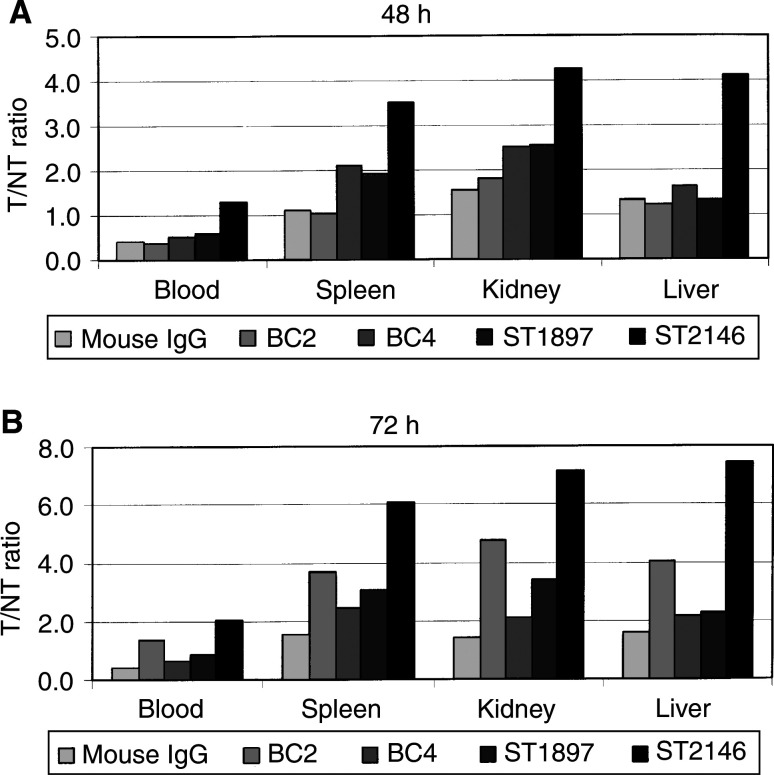
Tumour/nontumour ratios of ^125^I-labelled ST2146, BC4, BC2, ST1897 and normal mouse IgG at 48 and 72 h after administration.

To address the pharmacology behaviour of ST2146 and to evaluate its ability to localise at the tumour mass, comparative biodistribution studies of ^125^I-labelled ST2146, BC4, BC2 and ST1897 were performed in nude mice grafted with tenascin expressing human colon carcinoma HT29. The reactivity of the antitenascin antobodies on HT29 was preliminary confirmed by immunohistochemistry. The staining pattern that resulted was very similar for all antibodies tested while the isotype-matched controls were negative (data not shown). The immunoreactivity of radiolabelled antibodies evaluated by ELISA was found to be at least 80% that of each unlabelled one (data not shown). The results in Figure 7A

show that after 48 h from injection, the %i.d. g^−1^ of tumour of antitenascin Mabs ranges between 6 and 9% compared to 4% of the normal mouse IgG. After 72 h from injection, ST2146 showed approximately 16% of the i.d. g^−1^ of tumour compared to about 8% of either BC2 or ST1897 or less than 5% of BC4 antibodies. The highest concentration of ST2146 in the tumour, within the panel of antibodies tested at both 48 and 72 h from injection, is associated with the lowest amount on either blood, spleen, kidney or liver (Figure 7B–E). The highest tumour accumulation of ST2146 together with the lowest accumulation on nontarget tissues results in the most favourable tumour/nontumour (T/NT) ratios at both 48 and 72 h as shown in Figure 8A, B

and indicate that this Mab is the best at specific tumour targeting within the panel of antitenascin monoclonal antibodies tested in the present study. Particularly, at 72 h, the amount of ST2146 on the tumour is about twice the amount still present in the blood and more than seven times the amount present in liver, spleen and kidney. Additional biodistribution data produced with ST2146 biotinylated with different biotin/Mab ratios indicate that about 10–15% i.d. g^−1^ of tumour is maintained up to 10 days while the T/NT ratios increase up to three times *vs* the blood and more than 20 *vs* either liver, spleen or kidney independent of the extent of antibody biotinylation (manuscript in preparation).

## DISCUSSION

The main objective of pretargeting is to maximise the accumulation of drugs or radioisotopes at the tumour site while minimising distribution at nontarget organs. The potency and the selectivity of the monoclonal antibody are essential prerequisites for optimal pretargeting. The previously established monoclonal antibodies BC4 and BC2 proved to be useful for tumour targeting and pretargeting in several clinical trials. BC4 recognises an epitope within the EGF-like repeats of human tenascin. This epitope is shared by both small and large tenascin isoforms. BC2 recognises only the large tenascin being directed towards an epitope within the alternatively spliced A–D region which is absent in the small tenascin variant. Although Mabs that do not discriminate between small and large tenascin, like BC4, should more likely crossreact with normal tissues, previous immunohistochemical analysis of BC4 and BC2 reactivities on a large panel of normal tissues indicated a substantial similarity on the recognition patterns for the two antibodies (Natali *et al*, 1991), thus legitimating the idea that the crossreactivity of BC4 with normal tissue tenascin might be operationally overcome by the overexpression of tenascin of neoplastic tissues. In fact, biodistribution data from clinical trials showed that pretargeting with BC4 results in about 25 times more radioactivity on the tumour than on non-tumour tissues (Paganelli *et al*, 1999), while no biodistribution data are available for BC2. The good tumour/non tumour distribution ratio of BC4 in patients could be the result of either efficient chasing which in the three-step method reduces the nonspecific distribution, or could be because of overexpression of tenascin on the tumour compared to normal tissues, or both. On the other end, the frequency and level of expression of the tenascin epitopes derived from alternative splicing are not yet fully elucidated. In fact, a previous immunohistochemistry study, with two recombinant antitenascin antibodies, showed that 96 different tumours, including brain, breast and lung, were all strongly positive with the antibody recognising an epitope shared by small and large tenascins, while only 28 were positive with the other antibody recognising an epitope within the alternatively spliced region (Carnemolla *et al*, 1999). Taking into account clinical results with BC4 in the three-step method (Paganelli *et al*, 1999; Grana *et al*, 2002), we decided to generate a BC4-like antibody as a first priority while also pursuing the selection of a BC2-like antibody to better address the issue of optimal tenascin targeting in future studies. Advances in pretargeting biotechnology have been recently reviewed by Goodwin and Meares (2001). Besides the encouraging clinical results of Paganelli's group in brain tumours with the three-step method, others pursuing a two-step pretargeting method also obtained high efficacy in tumour therapy in the animal model (Axworthy *et al*, 2000) and encouraging clinical results in colon and lung cancers using a streptavidin–Mab conjugate (Breitz *et al*, 1999,^2000^; Knox *et al*, 2000). The two methods share the fundamental concept of using monoclonal antibodies for tumour pretargeting while differing in several aspects. The procedure to obtain the streptavidin–Mab conjugate, used in the two-step method, requires a chemical derivatisation of streptavidin, reduction and reoxidation of the Mab and the separation of the streptavidin–Mab conjugate from unconjugated streptavidin by chromatography. This procedure is more complex than the antibody biotinylation of the three-step method, which requires a simple chemical reaction followed by buffer exchange and which is therefore more suitable to industrial development. Moreover, speculating about the potency of the two conjugates, the biotinylated-Mab (three-step method) might be superior to the streptavidin–Mab (two-step method) as the several biotins on the antibody might allow the binding of more than the single avidin present in the conjugate of the two-step method. However, the three-step treatment is obviously more cumbersome to develop than the two-step one, requiring one additional step and one additional chasing that increases the products to be sequentially administered from three to five. In the present study, we found that the ST2146 monoclonal antibody exhibits higher immunoreactivity than BC4, BC2 and ST1897. The highest immunoreactivity of ST2146 is consistent with its homogeneity as opposed to the BC4 and BC2 nonhomogeneity and is associated with the highest affinity among the antibodies tested. The highest affinity of ST2146 also correlates with the highest *in vitro* binding capacity in condition of antigen limitation. This feature is particularly important in relation to the intended use of ST2146 as tumour pretargeting agent. In fact, previous animal studies from Zuckier *et al* (2000) indicated that a low-density antigen can be effectively targeted only by high-affinity antibodies. Our present data confirm that ST2146 exhibits the highest tumour-targeting capacity in nude mice xenograft models compared to three other monoclonal antitenascin antibodies, including BC2 and BC4. We believe that ST2146 might be a good candidate for further clinical development of PAGRIT.
